# Collaborative development of an electronic Personal Health Record for people with severe and enduring mental health problems

**DOI:** 10.1186/s12888-014-0305-9

**Published:** 2014-11-18

**Authors:** Liam Ennis, Dan Robotham, Mike Denis, Ninjeri Pandit, Dave Newton, Diana Rose, Til Wykes

**Affiliations:** King’s College London, Institute of Psychiatry, De Crespigny Park, London, SE5 8AF UK; South London and Maudsley NHS Foundation Trust, London, UK

**Keywords:** Personal health record, ePHR, Shared care, Patient empowerment, eHealth, Psychosis, Computer literacy

## Abstract

**Background:**

Previous attempts to implement electronic Personal Health Records (ePHRs) underline the importance of stakeholder involvement. We describe the development of an ePHR for people with severe and enduring mental health problems, and provide a model of involving stakeholders throughout.

**Methods:**

There were three stages to the development of the ePHR. These were 1) identifying and responding to user and clinical needs; 2) preliminary testing; and 3) preliminary implementation. Stakeholder involvement was pervasive in all stages. We collaborated with 133 stakeholders in the first stage, 13 in the second, and 26 in the third. On the micro-level, a service user researcher conducted much of the data collection and analysis. On the macro-level, a service user advisory group guided decisions throughout the project, and a service user was an active member of the project executive board and the implementation team.

**Results:**

Service users and clinicians preferred an interactive ePHR with features such as access to care plans and care notes, a mood tracker, patient reported outcomes feeding into the clinical record, and social networking features. Many of the above were constructed following consultation with the relevant professionals, however further consultation is required before building a social networking function or providing access to full care notes. Service users positively rated the usability of the ePHR. Drop-in sessions helped service users access technology and learn how to use the ePHR.

**Conclusions:**

We outline four considerations for future developers of ePHRs: appeal, construction, ease of use, and implementation. Success rests on implementation in routine practice, so ePHRs must be intuitive and useful for both service users and staff. Continued involvement of end users throughout the design and testing process can help to achieve this goal.

**Electronic supplementary material:**

The online version of this article (doi:10.1186/s12888-014-0305-9) contains supplementary material, which is available to authorized users.

## Background

Anyone who has used the National Health Service (NHS) within the UK has a clinical record detailing their medical history [1]. Traditionally, the clinician holds this record. The advent of electronic Personal Health Records (ePHRs) changes this [2]. An ePHR may contain some of the information held within the clinical record, but it is accessed and maintained by the patient [2]. Patients typically access ePHRs online, through a patient portal. A patient portal is a secure website through which patients can access their ePHR [3].

The introduction of ePHRs is a goal for UK Government [4], and England’s Department of Health has committed to providing patients with access to their primary care records online by 2015 [5]. There is a widespread belief that ePHRs will improve continuity of care [6,7], reduce medical errors [8], and allow patients to be better informed, thereby facilitating communication and empowerment [9]. Furthermore, ePHRs may benefit researchers, since they enable the collection of health outcomes data provided by the patient [10].

There are many examples of ePHRs in primary care [11], but there is sometimes reluctance to introduce them to secondary mental health services. This is often due to concerns of risk, confidentiality, and sensitivity of information [12]. However, for mental health service users these records could be especially useful. This is because mental health problems can be long-term, involve multiple service providers, and often coexist with other health complaints [13]. An ePHR could act as a hub to connect services, placing the patient at the centre [2].

We set out to develop an ePHR, designed with and for those with severe and enduring mental health problems. Our approach was informed by previous successes and failures. Both emphasise the inclusion of end users throughout the development process [14-16]. The current paper offers 1) a description of our ePHR; and 2) a model of service user inclusion in technology design and development.

## Methods and results

### Setting and participants

This ePHR was developed as part of the NIHR Biomedical Research Centre, a partnership between South London and Maudsley NHS Foundation Trust (SLaM) and the Institute of Psychiatry at King’s College London. All stages were conducted at sites within SLaM. An advisory board was set up to guide the project. Board members included service users, clinicians from psychosis services, clinicians from primary care services, members of the IT department within SLaM, and the Trust patient confidentiality guardian. The project team gained approval from an independent Research Ethics Committee (NRES Committee London - Camden & Islington, Reference 10/H0722/79). Much of the work was completed by researchers within the Service User Research Enterprise, which is a unit employing researchers who also have experience of using mental health services. Researchers therefore shared some of the same experiences as the participants. Written informed consent was obtained from all participants in research studies. Development consisted of three distinct stages, described in turn.

### Stage 1: Identifying needs and priorities

#### Procedure

This stage involved two procedures. First, interviews and focus groups were held with service users to discover whether there was an appetite for an ePHR within this population, and to ascertain which features people would like the ePHR to incorporate. Focus groups were recorded and transcribed. Second, we surveyed levels of computer literacy and access amongst service users in SLaM. This is because there is little information on readiness to use technology for people with psychosis, who may have had educational opportunities limited by the early onset of their symptoms [12,17].

Interview schedules and survey questions were developed collaboratively with a Service User Advisory Group. This was to ensure that the questions asked were relevant to this population, both in content and style.

#### Results

Findings relating to service users’ internet access and awareness have been published [18]. Briefly, surveys were conducted with 121 mental health service users living in South London. Findings suggested that people from black and other minority ethnic groups might need extra support when engaging with online health-related information, and also that mobile phones and computers were the most familiar devices for accessing the internet [18].

Separate focus groups were also conducted. Simple content analysis revealed that service users:

1. wanted more information about their care; “*…having like information about medication, the different ones and the side effects. I for one have definitely looked up every medication I’ve been on”*
2. were interested in monitoring their own health and wellbeing; *“…the thought process activity diaries, that’s something I was thinking of starting”*
3. had concerns about the sensitivity and security of information within the ePHR. One concern related to accessing the ePHR and subsequently forgetting to delete browsing history. Security concerns were particularly salient for paranoid service users; “*I’m not interested anymore … [computers] were reading my thoughts”*
4. wanted the ePHR to be connected and interactive, allowing them to contact their clinician and to communicate with others through social networking; *“maybe leave messages and so on you could post things on it … so you’d be like in a continuous dialogue”*
5. may have had problems with memory and organisation, thus preferring a simple, minimal interface.

##### System overview

**Figure 1:**
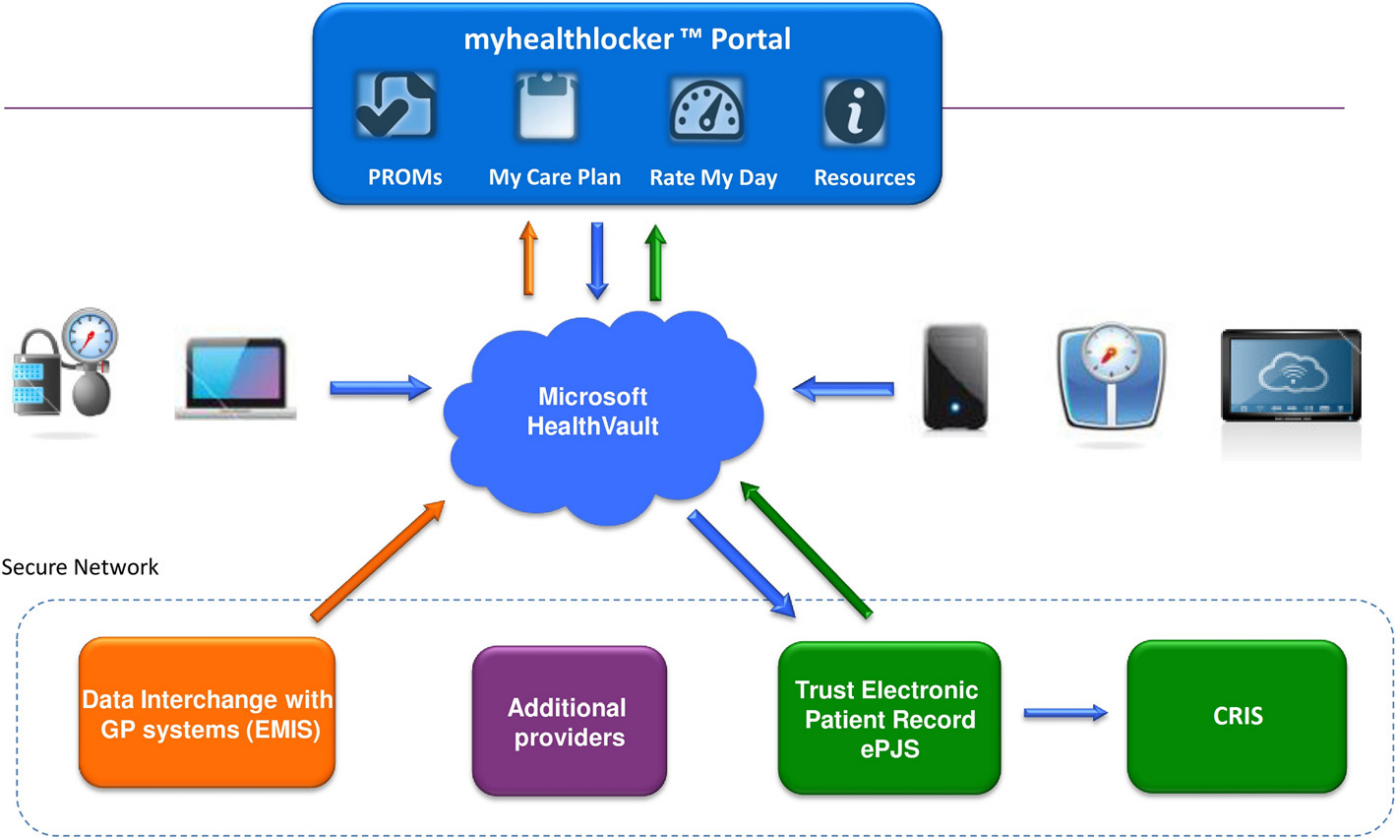
**Schematic showing the myhealthlocker™ ePHR.**

We named the ePHR we developed *myhealthlocker.* Figure 1 illustrates the myhealthlocker connected health model, in response to stage one scoping. The patient portal acts as the front end to Microsoft® HealthVault™. HealthVault is a secure online platform which enables the confidential collection, storage and sharing of health information. A two-way link was built between the patient portal and HealthVault, and between HealthVault and the Trust-held clinical record. This meant that information could be transferred from the patient portal to the Trust-held record, and vice versa, but only via HealthVault. This allowed interaction whilst maintaining the independent ownership of each party’s record. The two-way link between services and service users was a critical component of our ePHR. This infrastructure encourages dialogue and greater involvement of the service user in their care. A one-way link was established with EMIS, the most commonly used primary care records system in the UK. This meant that myhealthlocker users would be connected to both primary and secondary care services. Note that Figure 1 also shows a link to the Clinical and Research Interactive Search (CRIS) system, which allows researchers to search pseudonymised medical records [10]. Hence data entered by service users will contribute to the data available for audit to improve services and for clinical research. Security in the system was ensured because HealthVault undergoes continual security updates and extensive security testing. Another advantage of HealthVault is that all communication between HealthVault and other systems is encrypted, as well as the information contained within HealthVault itself (see http://msdn.microsoft.com/en-gb/healthvault/cc196394.aspx).

It was necessary to ensure that the model we developed was compliant with data protection and information governance regulations. We therefore consulted with information governance experts, and sought advice from SLaM Trust’s patient confidentiality guardian. UK information governance regulations are too lengthy for description here, but full details can be accessed from the Information Commissioner’s Office website [19].

Data ownership was a consideration outlined in discussions with the Trust’s patient confidentiality guardian. SLaM was identified as the data controller. Service users were identified as the ‘data subjects’ and the ultimate owners of the data.

In response to the results from our scoping exercises, the primary components to be included were care plans and Patient Reported Outcome Measures (PROMS), allowing service users to self-monitor their health. In addition myhealthlocker included customisable fields allowing users to create their own outcome measures (known as a ‘Rate My Day’ diary) in order to monitor health outcomes relevant to each service user. Common examples included sleep, diet, exercise, smoking and concentration.

The two-way link between the patient portal and the Trust-held record was tested by sending service users’ care plans from the clinical record to the patient portal. In the other direction, PROMs were completed by service users and automatically uploaded to the Trust-held record. The first PROM to be tested was the Warwick-Edinburgh Mental Well-Being Scale (WEMWBS). This is a 14-item, five-point Likert survey used for measuring mental wellbeing, and it is popular with service users [20].

Service users wished to have more information about how to monitor their health, so the ePHR included information on various physical health conditions. All text was written by service users, and reviewed by local service user groups. The ePHR focused on general health, rather than being specific to mental health, thus avoiding the perceived stigma associated with using the ePHR in public. Links to relevant organisations, resources such as NHS Choices, guides to legislation such as the Mental Health Act, and self-help information were added. The site therefore provided a signposting function.

### Stage 2: Preliminary acceptability testing

#### Procedure

Our two groups of end users (service users and clinicians) provided feedback on the beta version of myhealthlocker.

(i) *Service users.* Eight service users were recruited through a local register from various mental health services within SLaM. A service user researcher visited service users in their own homes and showed them the features of myhealthlocker. They were asked a series of structured questions about the attractiveness of the site, appropriateness of text, ease of navigation, usefulness of the ePHR, whether it was culturally relevant, and whether it was user friendly. Service users were asked to rate each domain on a scale of 1-5, to give an indication of any areas that were lacking. Service users were also asked for their comments, which were recorded. In total, each session lasted 1-2 hours. Service users were paid £15 for their time.
(ii) *Clinicians.* We gathered feedback from clinical staff in two outpatient teams for people with psychosis. Staff members within these teams were given a test password for myhealthlocker and asked to test the site for one week, after which they provided feedback to the project team. Five members of staff gave feedback.

#### Results

Table 1 shows mean acceptability scores for myhealthlocker.

**Table 1:** **Mean acceptability scores of myhealthlocker (N = 8)**

**Domain**	**Score**
Cultural appropriateness	4.6
Quality of written text	4.3
Ease of navigation	4.1
Site attractiveness	4.1
User friendliness	4.1
Perceived usefulness	3.9

Scores were rated on a 5-point likert scale: 1 = Very poor; 2 = Poor; 3 = Adequate; 4 = Good; 5 = Very good.

Whilst all measures were scored higher than “Adequate”, changes were made following the beta testing. The most substantial change related to the layout of the site, making it easier to navigate. To improve the perceived usefulness of myhealthlocker, a Body Mass Index calculator, an exercise diary, a blood pressure log, a blog function and information about medication and welfare benefits were all added.

Clinicians suggested adding the Worry Tree (a CBT tool providing a structured way of monitoring and assessing problems relating to anxiety; see Additional file 1: Figure S1), the Voices Diary (a motivational tool allowing service users to record auditory hallucinations), and audio files for CBT and mindfulness. These suggestions were incorporated. Furthermore, two PROMs were added, the Generalised Anxiety Disorder 7-item scale (GAD7; [21]) and Personal Health Questionnaire (PHQ; [22]). Both of these are popular self-report measures, commonly used in mental health settings. Whilst these measures were not chosen by service users, we felt that including them would improve the perceived utility of myhealthlocker for clinicians since they are routinely collected anyway. We hoped that this would encourage clinicians to engage with myhealthlocker and therefore take advantage of the connected healthcare model. Service users could complete these PROMs and view the results graphically.

### Stage 3: Preliminary implementation

#### Procedure

A service user planning committee was set up before making myhealthlocker available to a wider pool. This consisted of four service users. The purpose of the committee was to discuss ways in which myhealthlocker could be disseminated. Based on results from stage one, and advice from the service user planning committee, the project team developed weekly ‘drop-in’ sessions within local community venues. The purpose of these sessions was to provide service users with access to myhealthlocker, but also to give them the opportunity to use the internet and improve computer literacy. Some did not have access outside the drop-in session, and regular attendees were lent devices such as tablets or smartphones. Service users who were lent devices were also given an information sheet containing tips for online safety. Some service users with a high standard of computer literacy were offered volunteering opportunities at the drop-in. So far, three service users have acted as volunteer co-facilitators.

At the time of writing, 23 people attended drop-ins who had used psychosis services within the Trust. Each was asked to complete a 17-item feasibility questionnaire, covering demographic questions, the process of using the site for completing PROMs, setting-up and using the account, IT skills and confidence, the speed, design, and layout of the site. The survey was predominantly quantitative, including yes/no and 5-point Likert scales. There was also an open-ended question on how the site could be improved. Some participants wrote additional comments on the questionnaire form. Data were analysed using Fisher’s exact test, t-tests, and Spearman’s correlations.

#### Results

At each drop-in session, the facilitator encouraged service users to complete the WEMWBS PROM and to monitor health and wellbeing by viewing their results over time. Of the 23 attendees surveyed, most (57%, n = 13) had been using mental health services for more than six years. 13 people in the sample (56.5%) were male. The sample represented a fairly diverse range of ages: one person in the sample (4.3%) was aged 25-34; 11 (47.8%) were aged 35-44; 5 (21.7%) were aged 45-54 and six people (26.1%) were aged 55-64. Despite a majority reporting confidence in using computers prior to using myhealthlocker (61%; n = 14), many people needed training in basic computer skills such as using a mouse and navigating websites. This training was provided ad hoc by the drop-in session facilitators. Confidence with computers was not related to age or gender.

The average number of sessions attended was 19 (range = 0—63). Number of attendances was not related to age or to gender of the users. All but one of those attending had completed the WEMWBS. The remaining service user stopped engaging with myhealthlocker soon after signing up. Of those who completed the WEMWBS (n = 22), 19 had looked at the results it generated. Most service users reported little difficulty in completing the PROM, rating it a mean of 4.4 on a scale of 1-5 (where 1 is defined as ‘complicated’ and 5 as ‘simple’). Most found the questions relevant to them (mean = 4.0), and did not feel that the process of completing the PROM was time consuming (mean = 2.1, where a score of 1 was defined as ‘quick’ and 5 was defined as ‘slow’). There were no differences between the responses of men and women, and none of the ratings were related to age.

Service user opinions on the ePHR were canvassed at each drop-in. All but one said that they found the site useful, and 73% (17 out of 23) thought they would continue to use it in the future. Service users thought the layout of the site was clear (mean = 4.0, n = 19), had few problems with the font size (mean = 4.2, n = 20), with navigating the site (mean = 3.7, n = 20), or with understanding the content (mean = 4.2, n = 20). Each item was measured on a 5-point Likert scale with higher scores representing favourable responses. Again, there were no significant differences in ratings provided by men and women, and age was not related to the ratings. Qualitative comments referred to limitations of data visualisation; “the graph [is] a bit difficult to interpret”.

## Discussion

We have described our experiences of creating an ePHR for people with severe and enduring mental health problems, particularly psychosis. We recognised that ePHRs in this population are likely to require additional considerations [23], and therefore our research is relevant to anyone who wishes to design ePHRs for other marginalised groups. We have collaborated with end-users – both staff and service users – at all stages. We believe this to be the most comprehensive involvement of stakeholders in e-health intervention design to date. Our experience allows us to set out four key considerations for future developers.

1. Appeal
  Our ePHR, myhealthlocker, has demonstrated some appeal to service users, shown by the adequate scores for all measures. It is encouraging to note that the site holds similar appeal across genders and age ranges. However, wide-scale uptake remains a challenge, shown by the relatively low number of people engaging with the site at the time of writing. (It is worth noting, however, that slow uptake is a feature of many ePHRs [24]).
  Electronic applications (apps) represent one way in which uptake might be improved, because apps for people with psychosis have shown some popularity in US studies [14]. Social networking may also encourage people to use the site [25]Recently, interactive sites relating to health and wellbeing have enjoyed some success (e.g., Big White Wall, an online mental wellbeing service [26]), and electronically supported “buddying” schemes have been attempted within SLaM [27]. However, none of the above has operated within the context of an ePHR. The Kaiser Permanente (KP) website is a good example of a very well-used ePHR, and so we can learn from their successes. Some of the most used functions of the KP website are patient-doctor communication, viewing test results online, and scheduling appointments [28]. We are currently developing secure conference facilities for proxy communication with clinicians, and the facilities for appointment scheduling and viewing test results are nearly complete. We hope that these will significantly improve appeal.
2. Construction
  The concept of public data sharing is controversial, and the England’s National Health Service was recently forced to delay plans to develop a database of anonymous medical records [29]. A primary reason for this was concern about private data and privacy breaches. Such issues are particularly important to people who may suffer stigma and discrimination concerning their diagnosis [30].
  The technical and ethical processes of building a social networking function into myhealthlocker are unresolved. Since myhealthlocker links to the Trust-held clinical record, the healthcare provider is potentially accountable for content entered into myhealthlocker. One possible solution would involve third party developers to host the information generated via the social network, but security cannot be compromised. Another option would be to moderate content; this is the method used by Big White Wall [26]. This of course would mean employing someone around the clock to moderate the site use. We are currently holding conversations with information governance specialists in order to assess available options.
3. Ease of use
  People with severe and enduring mental health problems may lack experience and confidence with computers [18]. At present, drop-in sessions have helped educate potential users about basic IT skills, allowing the service user to explore myhealthlocker in a safe environment. However, the scalability of these drop-in sessions is limited. In order to implement the ePHR in routine practice, it needs to be intuitive for service users and clinical staff without supervision. And even with an intuitive system, a basic level of computer skills is required and we have found that this cannot always be assumed.
  Although our data suggest that myhealthlocker is easy to use, more data on site usage is needed. Clarity of navigation is paramount with this population, since people with experiences of psychosis often have accompanying cognitive problems [31]. Clarity of navigation is also critical with internet-based resources, as the probability of leaving a website is highest within the first ten seconds [32].
4. Implementation
  Implementing ePHRs in clinical practice will require a shift in attitudes [33]. The implementation of online banking suggests that adoption of technology depends on perceived usefulness and perceived ease of use [34]; this must be demonstrated to both service users and clinical staff. Furthermore, in secondary care, the ePHR requires acceptance from both primary care and secondary care clinicians. The interests of such stakeholders will need to be balanced. Previous reviews suggest negative attitudes amongst clinicians regarding shared care in mental health [12], so the implementation of ePHRs is a continuing challenge.
  Engaging clinicians is critical to implementation [35]. The ePHR has the potential to benefit clinicians through a reduction in administrative burden and an improved ability to monitor service users’ outcomes, but staff must identify these benefits for themselves or there is a danger of myhealthlocker being perceived as a top-down deployment and this can cause resistance [35]. Research shows that close collaboration with staff can allay resistance and improve utility, since clinical staff know the context in which the ePHR will be introduced [35]. Research also shows that staff are frequently apprehensive about using computers and may have limited skills [36,37]. Consequently, additional skills training for staff should also be considered.

## Conclusion

We believe that success with the four considerations outlined above – appeal, construction, ease of use and implementation – can be achieved through the involvement of end users, both clinicians and service users. The eventual success of myhealthlocker will depend upon embedding it within routine practice between service users and clinical staff. The ePHR must fit with service users’ self-management practices, and be useful enough for clinicians to wish to engage service users with it. We have described the various stages of development of myhealthlocker, and the ways in which we included stakeholders at each stage. We believe that this involvement has allowed myhealthlocker to develop in accordance with the needs of service users and clinicians.

## Additional file

Additional file 1: Figure S1. An example of a “worry tree”, a CBT tool used to treat anxiety.

## References

[1] BCS The Chartered Institute for IT: **Keeping your online health and social care records safe and secure.** 2013. Downloaded from http://www.bcs.org/upload/pdf/guidance-health-and-socal-records.pdf, 04.09.2014.

[2] London Connect: **It's your record: a guide to accessing health records online.** 2012. Downloaded from http://www.myhealth.london.nhs.uk/sites/default/files/u3246/ItsYourRecord.pdf, 04.09.2014.

[3] National Network of Libraries of Medicine: **Personal Health Records (PHR) and Patient Portals.** http://guides.nnlm.gov/content.php?pid=287967&sid=2954062 accessed on 04.09.2014.

[4] Blumenthal D (2009). Stimulating the adoption of health information technology. New Engl J Med.

[5] Department of Health (2012). The Power of Information: Putting all of us in Control of the Health and Care Information we Need.

[6] Burns T, Catty J, White S, Clement S, Ellis G, Jones IR, Lissouba P, McLaren S, Rose D, Wykes T (2009). Continuity of care in mental health: understanding and measuring a complex phenomenon. Psychol Med.

[7] Jones DA, Shipman JP, Plaut DA, Selden CR (2010). Characteristics of personal health records: findings of the Medical Library Association/National Library of Medicine Joint Electronic Personal Health Record Task Force. J Med Libr Assoc.

[8] Hillestad R, Bigelow J, Bower A, Girosi F, Meili R, Scoville R, Taylor R (2005). Can electronic medical record systems transform health care? Potential health benefits, savings, and costs. Health Affair.

[9] Ueckert F, Goerz M, Ataian M, Tessmann S, Prokosch HU (2003). Empowerment of patients and communication with health care professionals through an electronic health record. Int J Med Inform.

[10] Stewart R, Soremekun M, Perera G, Broadbent M, Callard F, Denis M, Hotopf M, Thornicroft G, Lovestone S (2009). The South London and Maudsley NHS foundation trust biomedical research centre (SLAM BRC) case register: development and descriptive data. BMC Psychiatry.

[11] Pagliari C, Detmer D, Singleton P (2007). Potential of electronic personal health records. BMJ.

[12] Ennis L, Rose D, Callard F, Denis M, Wykes T (2011). Rapid progress or lengthy process? Electronic personal health records in mental health. BMC Psychiatry.

[13] Barnett K, Mercer SW, Norbury M, Watt G, Wyke S, Guthrie B (2012). Epidemiology of multimorbidity and implications for health care, research, and medical education: a cross-sectional study. Lancet.

[14] Ben-Zeev D, Brenner CJ, Begale M, Duffecy J, Mohr DC, Mueser KT: **Feasibility, acceptability, and preliminary efficacy of a smartphone intervention for schizophrenia.** *Schizophr Bull* 2014. doi:10.1093/schbul/sbu033. 10.1093/schbul/sbu033 PMC4193714 24609454

[15] Ben-Zeev D, Kaiser SM, Brenner CJ, Begale M, Duffecy J, Mohr DC (2013). Development and usability testing of FOCUS: a smartphone system for self-management of schizophrenia. Psych Rehabil J.

[16] Leonard KJ (2004). The role of patients in designing health information systems: the case of applying simulation techniques to design an electronic patient record (EPR) interface. Health Care Manage Sci.

[17] Kessler RC, Foster CL, Saunders WB, Stang PE (1995). Social consequences of psychiatric disorders, I: Educational attainment. Am J Psychiatr.

[18] Ennis L, Rose D, Denis M, Pandit N, Wykes T (2012). Can't surf, won't surf: the digital divide in mental health. J Ment Health.

[19] Information Commissioner’s Office: **Data protection.** http://ico.org.uk/for_organisations/data_protection accessed on 04.09.2014.

[20] Crawford MJ, Robotham D, Thana L, Patterson S, Weaver T, Barber R, Wykes T, Rose D (2011). Selecting outcome measures in mental health: the views of service users. J Ment Health.

[21] Spitzer RL, Kroenke K, Williams JB, Löwe B (2006). A brief measure for assessing generalized anxiety disorder: the GAD-7. Arch Intern Med.

[22] Kroenke K, Spitzer RL, Williams JB (2001). The Phq‐9. J Gen Intern Med.

[23] Druss BG, Ji X, Glick G, Silke A (2014). Randomized trial of an electronic personal health record for patients with serious mental illnesses. Am J Psychiatr.

[24] Wynia M, Dunn K (2010). Dreams and nightmares: practical and ethical issues for patients and physicians using personal health records. J Law Med Ethics.

[25] Arguello J, Butler BS, Joyce E, Kraut R, Ling KS, Rosé C, Wang X (2006). Talk to me: foundations for successful individual-group interactions in online communities. Proceedings of the SIGCHI conference on Human Factors in computing systems.

[26] Big White Wall: **Big White Wall.** http://www.bigwhitewall.com/ accessed on 04.09.2014.

[27] Drake G, Csipke E, Wykes T (2013). Assessing your mood online: acceptability and use of Moodscope. Psychol Med.

[28] Silvestre A-L, Sue VM, Allen JY (2009). If you build it, will they come? The Kaiser Permanente model of online health care. Health Aff.

[29] Triggle N: **Giant NHS database rollout delayed.** In *BBC News.* BBC News Health: BBC News; 2014 (online article; http://www.bbc.co.uk/news/health-26239532).

[30] Dinos S, Stevens S, Serfaty M, Weich S, King M (2004). Stigma: the feelings and experiences of 46 people with mental illness Qualitative study. Br J Psychiatry.

[31] O'Carroll R (2000). Cognitive impairment in schizophrenia. Adv Psychiatr Treat.

[32] Liu C, White RW, Dumais S (2010). Understanding web browsing behaviors through Weibull analysis of dwell time. Proceedings of the 33rd International ACM SIGIR Conference on Research and Development in Information Retrieval.

[33] Pagliari C, Detmer D, Singleton P (2007). Electronic Personal Health Records.

[34] Chau PYK, Lai VSK (2003). An empirical investigation of the determinants of user acceptance of Internet banking. J Org Comp Elect Com.

[35] Cresswell K, Sheikh A (2009). The NHS Care Record Service (NHS CRS): recommendations from the literature on successful implementation and adoption. Inform Primary Care.

[36] Kirshbaum MN (2004). Are we ready for the electronic patient record? Attitudes and perceptions of staff from two NHS trust hospitals. Health Inform J.

[37] Ward R, Stevens C, Brentnall P, Briddon J (2008). The attitudes of health care staff to information technology: a comprehensive review of the research literature. Health Inform Lib J.

