# Supplementary material for: Collaborative development of an electronic Personal Health Record for people with severe and enduring mental health problems
Source: BMC Psychiatry. 2014 Nov 18;14:305. doi: 10.1186/s12888-014-0305-9 (PMC4245793; doi:10.1186/s12888-014-0305-9)

THE WORRY TREE

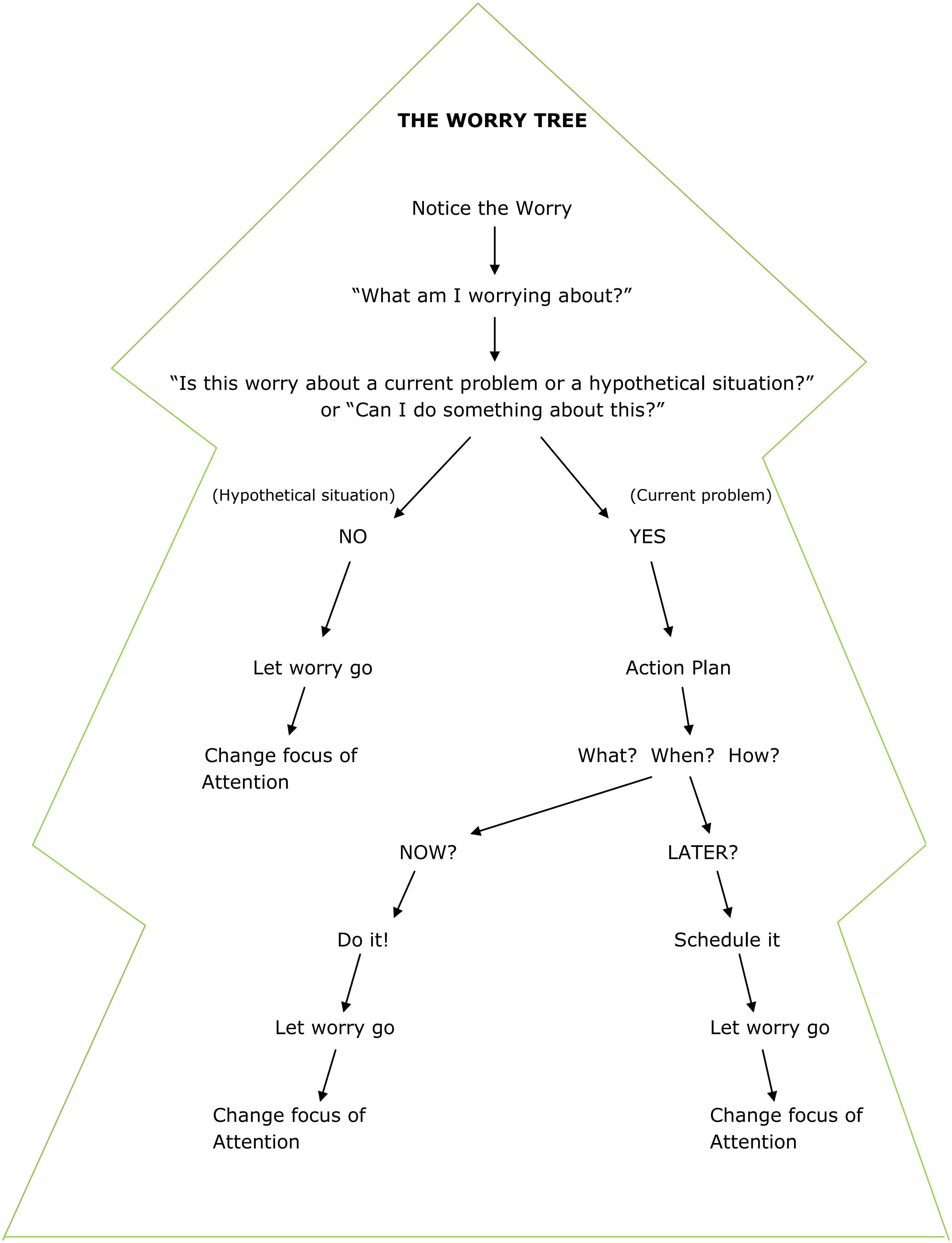

Supplement: Additional file 1: Figure S1. — An example of a “worry tree”, a CBT tool used to treat anxiety. [file 12888_2014_305_MOESM1_ESM.pdf]
